# Modulation of SLFN11 induces changes in DNA Damage response in breast cancer

**DOI:** 10.1186/s12935-023-03144-w

**Published:** 2023-11-24

**Authors:** Christophe Michel Raynaud, Eiman I. Ahmed, Ayesha Jabeen, Apryl Sanchez, Shimaa Sherif, Tatiana C. Carneiro-Lobo, Amany Awad, Dina Awartani, Adviti Naik, Remy Thomas, Julie Decock, Gabriele Zoppoli, Davide Bedongnetti, Wouter R. L. Hendrickx

**Affiliations:** 1 Tumor Biology and Immunology Lab, Research Branch, Sidra Medicine, Doha, Qatar; 2 Department of Biomedical Science, College of Health Sciences, Qatar University, Doha, Qatar; 3 Translational Cancer and Immunity Center, Qatar Biomedical Research Center, Doha, Qatar; 4 College of Health and Life Sciences (CHLS), Hamad Bin Khalifa University (HBKU), Doha, Qatar; 5 Department of Internal Medicine (DiMI), University of Genoa and Ospedale Policlinico San Martino, Genoa, Italy; 6 Ospedale Policlinico San Martino IRCCS per l’Oncologia, Genoa, Italy; 7 Clinical and Experimental Oncology and Hematology, Ospedale Policlinico San Martino, Genoa, Italy

**Keywords:** SLFN11, Breast cancer, Chemosensitivity, UNISAM, KRAB, Cisplatin, Epirubicin, Olaparib

## Abstract

**Background:**

Lack of Schlafen family member 11 (SLFN11) expression has been recently identified as a dominant genomic determinant of response to DNA damaging agents in numerous cancer types. Thus, several strategies aimed at increasing SLFN11 are explored to restore chemosensitivity of refractory cancers. In this study, we examined various approaches to elevate SLFN11 expression in breast cancer cellular models and confirmed a corresponding increase in chemosensitivity with using the most successful efficient one. As oncogenic transcriptomic downregulation is often driven by methylation of the promotor region, we explore the demethylation effect of 5-aza-2′-deoxycytidine (decitabine), on the SLFN11 gene. Since SLFN11 has been reported as an interferon inducible gene, and interferon is secreted during an active anti-tumor immune response, we investigated the in vitro effect of IFN-γ on SLFN11 expression in breast cancer cell lines. As a secondary approach to pick up cross talk between immune cells and SLFN11 expression we used indirect co-culture of breast cancer cells with activated PBMCs and evaluated if this can drive SLFN11 upregulation. Finally, as a definitive and specific way to modulate SLFN11 expression we implemented SLFN11 dCas9 (dead CRISPR associated protein 9) systems to specifically increase or decrease SLFN11 expression.

**Results:**

After confirming the previously reported correlation between methylation of SLFN11 promoter and its expression across multiple cell lines, we showed in-vitro that decitabine and IFN-γ could increase moderately the expression of SLFN11 in both BT-549 and T47D cell lines. The use of a CRISPR-dCas9 UNISAM and KRAB system could increase or decrease SLFN11 expression significantly (up to fivefold), stably and specifically in BT-549 and T47D cancer cell lines. We then used the modified cell lines to quantify the alteration in chemo sensitivity of those cells to treatment with DNA Damaging Agents (DDAs) such as Cisplatin and Epirubicin or DNA Damage Response (DDRs) drugs like Olaparib. RNAseq was used to elucidate the mechanisms of action affected by the alteration in SLFN11 expression. In cell lines with robust SLFN11 promoter methylation such as MDA-MB-231, no SLFN11 expression could be induced by any approach.

**Conclusion:**

To our knowledge this is the first report of the stable non-lethal increase of SLFN11 expression in a cancer cell line. Our results show that induction of SLFN11 expression can enhance DDA and DDR sensitivity in breast cancer cells and dCas9 systems may represent a novel approach to increase SLFN11 and achieve higher sensitivity to chemotherapeutic agents, improving outcome or decreasing required drug concentrations. SLFN11-targeting therapies might be explored pre-clinically to develop personalized approaches.

**Supplementary Information:**

The online version contains supplementary material available at 10.1186/s12935-023-03144-w.

## Background

Schlafen 11 (SLFN11), is a highly conserved mammalian nuclear protein found to be an essential component during replication stress. In brief, SLFN11 induces an irreversible replication block and eventually apoptosis [1]. SLFN11 binds to Replication Protein A (RPA) in the stalled replication forks. It interacts with MCM3 (Minichromosomal maintenance complex component 3), and DHX9 (DExH-box helicase) [2, 3]. This leads to the opening of the chromatin around the replication initiation sites, which activates the transcription of immediate early genes that can induce cell cycle arrest. Thereby blocking any further replications from occurring [4]. This is done independently of, and in parallel with, the ATR-CHK 1 S-phase checkpoint in the DDR pathway [2].

SLFN11 expression has been strongly correlated with sensitivity to DNA damaging agents (DDA), like platinum salts such as cisplatin, or agents affecting DNA Damage Repair (DDR) like Poly (adenosine diphosphate-ribose) polymerase inhibitor (PARPi) such as Olaparib and to topoisomerase I and II inhibitors such as topotecan and Epirubicin [5, 6]. More recently SLFN11 immunohistochemistry of ovarian cancer tissue was able to predict response to platinum-based chemotherapies [7]. Higher SLFN11 gene expression showed a better prognosis in breast cancer [8]. Hence, SLFN11 could make for a good predictive biomarker of therapeutic response or a treatment target in many cancers including breast cancer.

Next to a predictive biomarker SLFN11 could be a therapeutic target for sensitizing cancer cell to chemo, radio, or immunotherapy. To comprehensively quantify the effect of the gene’s re-induction on the cells chemosensitivity to different drugs we modulate the SLFN11 expression in several ways.

The SLFN11 gene expression can be suppressed by three epigenetic mechanisms: promotor methylation [3], histone deacetylation [9], and histone methylation by the Polycomb Repressive Complex (PRC) [10]. Most cell lines that lack SLFN11 expression were found to feature hypermethylation-associated silencing in the CpG island in the promotor region of the SLFN11 gene. This silencing correlates with increased resistance to platinum chemotherapies drugs [3]. This prompted us to attempt demethylation of the promotor region of SLFN1 using decitabine.

In addition, SLFN11 has been identified as an interferon (IFN)-stimulated gene [11]. SLFN11 gene expression is induced upon interferon signaling in case of viral infections such as HIV or Zika virus [12]. Therefore, we tried inducing SLFN11 expression using in-vitro Interferon Gamma stimulation in our breast cancer cells.

This paper comprehensively investigates the relationship between modulation of SLFN11 expression either by interferon, decitabine (DAC) demethylation, co-culture with activated PBMC’s releasing IFN or CRISPR alteration and the resulting changes in chemosensitivity. Hypothesizing that in cancers which are resistant to chemotherapy, upregulation of SLFN11 can restore their chemosensitivity. Once cell lines were selected of the multiple methods investigated to upregulate SLFN11 expression, the most robust modulation was achieved with CRISPR, which was precise, effective, and lacked wider transcriptomic side effects.

## Methods

### Cell lines and culture

All cell lines were purchased from ATCC (Table 1). All cell lines were cultured in advanced RPMI (Gibco, #12633012) complemented with 10% FBS (Sigma, #F4135), Glutamax (Gibco, #35050061) and antibiotic–antimycotic (Gibco, #15240096). Cells were cultured at 37 °C, 5% CO2 and 95% humidity. Cells were detached using TrypLE express enzyme (Gibco, #12605036). Human foreskin fibroblasts were used across the manuscript as positive control and reference for expression of SLFN11.

**Table 1:** Cell lines

Cell line name	Catalogue number	Tissue of origin	Disease	BRCA mutations
HFF	90011887	Human Foreskin Fibroblast	HPRT-derivative of the SV40 transformed human fibroblast line GM0637	None
BT-549	HTB-122	Breast; Mammary gland	Carcinoma; Ductal; ER−	none
T47D	HTB-133	Breast; Mammary gland	Carcinoma; Ductal; ER+	None
MDA-MB-231	HTB-26	Breast; Mammary gland	Adenocarcinoma; ER−	None
MDA-MB-436	HTB-130	Breast; Mammary gland	Adenocarcinoma; ER−	BRCA1
MDA-MB-468	HTB-132	Breast; Mammary gland	Adenocarcinoma; ER−	BRCA2
MCF-7	HTB-22	Breast; Mammary gland	Adenocarcinoma; ER +	None
MDA-MB-453	HTB-131	Breast; Mammary gland	Carcinoma; Metastatic; ER-	None
HCC70	CRL-2315	Breast; Duct; Mammary gland	Carcinoma; Ductal; TNM stage IIIA, grade 3; ER−	None

### DNA/RNA extraction

DNA was isolated using DNeasy blood and tissue kit (Qiagen, #69506) according to manufacturer recommendations. DNA was recovered in 100 μl of AE buffer and stored at − 80 °C until use. RNA was extracted using RNeasy mini kit (Qiagen, #74104) according to manufacturer recommendations with some modifications. Briefly, cell pellets were dissolved in 700 µl of Trizol LS reagent (Invitrogen, #10296028). Next, 200 µl of chloroform (Sigma, #34854) was added followed by extensive vortexing for 1 min and centrifugation for 15 min at 17.000*g* for 15 min. Supernatant was recovered and 750 µl of RLT buffer and 500 µl of Ethanol (Acros organics, #61509.0010) were added. After mixing, the sample was loaded on the MinElute column and centrifuged for 1 min at full speed, and this step was repeated until the whole sample passed through the column. The sample was washed with 500 µl of RPE and 500 µl of 80% ethanol before drying by centrifugation for 5 min at 17.000*g*. RNA was recovered in 16 µl of RNase free water and measured with Nanodrop 8000. RNA was stored at − 80 °C until use.

### Q-RT-PCR

1 µg of RNA was used for reverse transcription using TaqMan™ Reverse Transcription Reagents (Invitrogen, #N8080234) and random hexamers. cDNA was diluted 20 times with DNA/RNA free water. TaqMan™ Gene Expression Master Mix (Applied bioscience, #4369016) and Hs03003631_g1 (for Eukaryotic 18S rRNA) and Hs00536981_m1 (for SLFN11) TaqMan probes (Thermo scientific, #4331182) were used according to manufacturer recommendation. Quantitative Real time PCR (Q-RT-PCR) was run in 96 well plates on QuantStudio 12K flex system (Thermofisher Scientific). Q-RT-PCR was done in triplicate for each sample and data were analyzed by gene expression comparison using ΔΔCT on (QuantStudio 12K Flex Realtime PCR system V1.2.2) using S18 as the housekeeping gene.

### Western blot using capillary western blot

After culture, 5 × 10^6^ cells were washed with DPBS and lysed with 400 µl of RIPA Lysis and Extraction Buffer (Thermo Scientific, #89900) complemented with Halt™ Protease Inhibitor Cocktail (Thermo scientific, #78430) and sonication for 30 s. Cell debris was removed by 30 min centrifugation at 14.000*g*. Supernatants containing protein extract were kept at − 20 °C until use. Protein concentration was assessed using Pierce BCA protein assay kit (Thermo scientific, #23225). Capillary western blot (CWB) was done using a WES system (protein simple) with 12–230 kDa Separation Module, 8 × 25 capillary cartridges (Protein simple, #SW-W004), EZ Standard Pack 2 (Protein simple, #PS-ST02EZ-8) and Anti-mouse detection module (Protein simple DM-002). Mouse anti human SLFN11 (Santa Cruz, #SC-515071) and anti β-actin (Licor, #926-42212) both diluted at 1 in 100 were used as primary antibody.

Analysis was done using compass for Simple western (ProteinSimple, V5.0.0) (https://www.bio-techne.com/resources/instrument-software-download-center?filters%5Binstrument_category%5D%5B0%5D=372) and area of histogram peaks were used for quantification. All western blot analysis were normalized for β-actin expression. Raw files for each CWB presented are provided as supplementary data on FigShare (https://doi.org/10.6084/m9.figshare.22776191).

### Promoter methylation analysis by MSP

Promoter methylation was analyzed using Methyl Specific Polymerase Chain Reaction (MSP). Genomic DNA was extracted as previously described, bisulfite conversion was performed using EZ DNA methylation kit (Zymo research, # D5001). PCR was performed using the primers: Forward Methylated specific primer (GTAGCGGGGTAGAAAAGTAGAAC) and Reverse Methylated specific primer (TAAAATTTAACGACGACCGATACG) for methylated specific PCR with a PCR product of 108 bp. Forward Unmethylated specific primer (GTAGTGGGGTAGAAAAGTAGAAT) and Reverse unmethylated specific primer (TAAAATTTAACAACAACCAATACA) for unmethylated specific PCR with a PCR product of 105 bp, 1 µl of converted DNA and AmpliTaq Gold™ 360 Master Mix (Applied Biosystems, #4398876).

The PCR product was then run on 2% agarose (Sigma, #A4718) gel containing Ethidium Bromide (Sigma, #E1510) and picture were taken using Chemidoc XRS system (Biorad, # 1708265) with the single channel ethidium bromide agarose gel protocol. Band intensity was measured using Image J (https://imagej.nih.gov/ij/, 1997–2018. Schneider, C.A., Rasband, W.S., Eliceiri, K.W. "NIH Image to ImageJ: 25 years of image analysis") and relative intensity between methylated and unmethylated specific PCR was calculated.

### Drugs

5-Azacytidine (Decitabine) (DAC) (MP Biomedicals, #154803) was reconstituted at 20 mM in DMSO (Sigma, #D4540) and used at 5 µM final concentration; IFN-γ (Peprotech, #300-02-100UG) was diluted in water at 10 μM and used at 5 nM final concentration, cis-Diamineplatinium (II) dichloride (Cisplatin) (Sigma, #479306) was reconstituted fresh at 2 mM in NaCl solution 0.9% (Sigma, #SW8776) and used at various concentration as indicated; Epirubicin (Sigma,# 1237382) was reconstituted at 3.5 mM in water and used at various concentration; Olaparib (Biovision, #1952-25) was reconstituted at 57.54 mM in DMSO and used at various concentration.

### Indirect co-culture model

A total of 5 × 10^4^ cancer cells (BT-549, T47D and MDA-MB-231) were seeded per well in a 24‐well plate and cultured overnight. Total PBMCs were plated in a flat‐bottom multi‐well plate (Thermo Fisher Scientific, Nunclon Δ Surface) and incubated for 2 h at 37 °C and 5% CO_2_ and following the incubation, the non‐adherent peripheral blood lymphocyte population (PBLs) were isolated. Next, the non‐adherent PBLs were activated overnight using 2 μg/ml of plate‐bound anti‐human CD3 and CD28 antibodies (eBioscience) at 37 °C and 5% CO_2_. To set up the indirect co-culture, the activated PBLs were placed on top using transwell inserts with 0.4 µm pore size (Corning) and a Target:Effector (T:E) ratio of 1:20 to enable exchange of soluble factors between cancer cells and PBLs without direct cell‐cell contact. Cancer cells were plated alone without PBLs as control. The cells were co‐cultured for 72 h at 37 °C and 5% CO2, after which RNA was isolated from cancer cells for downstream analysis.

### CRISPR cell engineering—gRNA design

IDT custom gRNA design tool was used to design gRNA along the core region of the promoter, distributed along the promoter as illustrated in Additional file 1: Fig. S1.

The sequence of the gRNA is as follow (Table 2).

**Table 2:** gRNA sequence

gRNA number	gRNA sequence	On target score	Off target score
N1	TAGTATATAAGGACTCGACC	84	87
N2	GAAGGCCACTGAGTGCACCT	78	44
N4	AGGCCCACTTCTCACTGATG	74	47
N5	AATACACGTGCTACCCCAGA	73	73
N6	TGGGCTAGACCCTGAAGCAC	73	45
N7	ACACTCGGACAGAATCCTGG	72	68
N10	GAAGGAAACGGCCACCCCGT	66	84

The sequence of the scramble (SCR) gRNA used is GCACTACCAGAGCTAACTCA

Forward and reverse primers were ordered accordingly to be inserted in the appropriate plasmids as described below.

### CRISPR cell engineering—cloning of gRNA into UNISAM and KRAB plasmids

PB-UniSAM containing mCherry was a gift from Lesley Forrester (Addgene plasmid # 99,866; http://n2t.net/addgene:99866; RRID: Addgene_99866) [13]. pLV hU6-sgRNA hUbC-dCas9-KRAB-T2a-GFP was a gift from Charles Gersbach (Addgene plasmid # 71,237; http://n2t.net/addgene:71237; RRID: Addgene_71237) [14]. Cloning in PB-UniSAM was done using BbsI (New England Biolabs, #R3539L) as indicated in [13]. Cloning into pLV hU6-sgRNA hUbC-dCas9-KRAB-T2a-GFP was performed using BsmBI restriction enzyme (New England Biolabs, #R0580L) as indicated in [14]. Briefly, gRNAs were obtained from Integrated DNA technology as two single strand oligos and annealed in 40  μl of annealing buffer (Origene, #GE100007) with 2  μl of each oligo (100 μM) and annealed in the thermal cycler at 95  °C for 4 min followed by cooling to 25  °C with 1 °C/minute ramp. Annealed oligos were then diluted 10 times in water. 10 ng of purified *BbsI* linearized UniSAM or BsmBI linearized pLV hU6-sgRNA hUbC-dCas9-KRAB-T2a-GFP was ligated with 1  μl of diluted annealed gRNA with 0.5 μl of T4 ligase (New England Biolabs, #M0202L) in a total volume of 10 μl. Solution was incubated at room temperature for 2 h prior to transformation in *E. coli* STBL3 (Invitrogen, # C737303). Minipreps were performed using QIAprep Spin Miniprep Kit (Qiagen, #27106X4). For stable modification of cells using UniSAM, co-transfection was performed with pcDNA3-transposase gifted by Dr. Juan Cadinanos. Correctly assembled UniSAM and pLV hU6-sgRNA hUbC-dCas9-KRAB-T2a-GFP vectors were confirmed by complete Sanger sequencing using BigDye™ Terminator v3.1 Cycle Sequencing Kit (Applied Biosystem, #4337455) and DyeEx 2.0 Spin kit (Qiagen, #63204) according to manufacturer recommendation and analyzed on ABI3500xL (Applied biosystem, #4406016).

### CRISPR cell engineering—cell transfection

Electroporation was performed using Neon transfection system (Invitrogen, #MPK5000) with Neon™ Transfection System 10 µl Kit (Invitrogen, #MPK1096) using 2 µg of DNA for 1.10^5^ cells in 24 well plate. Electroporation protocol for each cell line was identified using pmaxCloningTM vector (Lonza, # VDC-1040). The optimal protocol for each cell line is indicated in Table 3.

**Table 3:** Electroporation conditions for each cell line

Cell line	Voltage	Width	Pulses
BT-549	1300	30	1
T47D	1400	30	1
MDA-MB-231	1600	20	1

After 1 week of culture, cells were further purified by sort

### CRISPR cell engineering—sort of cells

Cells were harvested and blocked in PBS with 5%FBS and 1%BSA and cell clumps removed on 40 µM cell strainer (Falcon, #382235). Single-cell suspension was analyzed and sorted on SORP FACSAriaIII (BD Biosciences Special Order Research Product). Data were processed with BD FACSDiva™ Software V8.0.1 (BD Biosciences). GFP fluorescence was acquired with 488 nm blue laser and 530/30 nm emission filter, and mCherry fluorescence was acquired with 561 nm yellow-green laser and 610/20 nm emission filter. During cell-sorting 4-way purity-phase mask was applied. To ensure maximum purity, cells were serially sorted 3 times prior analysis and use.

### Viability analysis

Cells were grown in 96 well plate for 24 to 72 h accordingly with or without treatment. 3000 and 5000 cells were plated for BT-549 and T47D respectively 24 h prior treatment. Viability was assessed using ATPlite Luminescence Assay System (Perkinelmer, #6016949) and Calcein AM (Invitrogen, #C3099). Luminescence was measured with Ensight plate reader (Perkinelmer, #HH34000000). Calcein AM was assessed by fluorescence intensity measurement by well scan from bottom with excitation at 494 nm and emission at 517 nm on Ensight plate reader (Perkinelmer, #HH34000000).

### mRNA sequencing

mRNA-sequencing was performed using QuantSeq 3’ mRNA-Seq Library Prep Kit FWD for Illumina (Cat. 015.96) (75 single-end) with a read depth of average 9.31 M, and average read alignment of 55.8%. Single samples were sequenced across multiple lanes, and the resulting FASTQ files were merged by sample. All samples passed FastQC (v. 0.11.8) were aligned to the reference genome GRChg38 using STAR (v. 2.6.1d) [15]. BAM files were converted to a raw counts expression matrix using HTSeq-count (v. 0.9.1) [16].

### RNAseq data normalization

Normalization was done using R Bioconductor package EDAseq (Exploratory Data Analysis and Normalization for RNA-Seq) (v. 2.28.0) [17] to remove within and between lane effects. Data was then quantile normalized using R Bioconductor package preprocessCore package (v. 1.56.0) [18] and log2 transformed. All downstream analysis was done using R (v. 4.1.2). Batch effects were removed for each cell line separately using ComBat () function from R Bioconductor package sva (v. 3.42.0) [19]. Genes with row sum equal to zero were removed before applying ComBat. Data were then combined, and quantile normalized again as described previously. Principal component analysis (PCA) was done based on genes expression to assess global transcriptional differences between the samples using prcomp function and plotted using R CRAN package ggplot2 (v. 3.3.5) [20]. For BT-549 CTRL and modified cells (UNISAM and KRAB) N = 6 biological replicates were sequenced and used for analysis. For T47D CTRL and modified cells (UNISAM and KRAB) N = 5 biological replicates were sequenced and used for analysis. For RNA expression after 72 h of cisplatin treatment, each cell line presented was sequenced as 9 biological replicates.

### Differentially expressed genes

Differentially expressed genes (DEGs) analysis was performed on log2 normalized mRNA expression data using R Bioconductor package limma (v. 3.50.0) [21] with Benjamini-Hochberg (B-H) FDR. Within each comparative analysis, genes with row sum equal to zero were removed. To visualize the overlap of differentially expressed genes between the conditions, R CRAN package VennDiagram (v. 1.7.1) was used [22]. Differentially expressed genes were then plotted in a heatmap using R Bioconductor package ComplexHeatmap (v. 2.10.0) [23].

### Single sample gene set enrichment analysis (ssGSEA)

ssGSEA was done using normalized, log2 transformed data for the selected list of genes. Enrichment score (ES) was calculated using gsva function from R Bioconductor package GSVA (v. 1.42.0) [24]. ES was calculated for genes obtained from DEG analysis using Limma. Gene sets to reflect enrichment of apoptosis and glycogen metabolism pathways were downloaded from Molecular Signatures Database (MSigDB) [25].

### Pathway enrichment analysis

For enriched pathway analysis, list of differentially expressed genes (FDR < 0.01, LogFC >  = 1) was uploaded to Ingenuity Pathway Analysis (IPA). Pathways data were exported from IPA as excel file and used to regenerate the figure using R CRAN package “ggplot2 v. 3.3.5”.

### Statistical analysis

For statistical analysis and graphical presentation, Excel (Microsoft Corporation) and Graphpad prism V9.3.1 (Domatics) software was used. Numerical results are given as means ± SD (N = sample size). The statistical significance for CWB and Q-PCR was assessed with Graphpad with unpaired Student’s t test. The statistical significance for the comparison of genes expression and enrichment score was calculated using unpaired t test using R programming function “stat_compare_means” from ggpubr package. Statistical significance was accepted for *p < 0.05; **p < 0.01; ***p < 0.001; ****p < 0.0001. Pearson's correlation coefficient was employed for calculating the correlation between Q-PCR, CWB, and methylation data.

## Results

### Baseline SLFN11 expression and associated methylation profiles across a panel of 8 different breast cancer cell lines

**Fig. 1:**
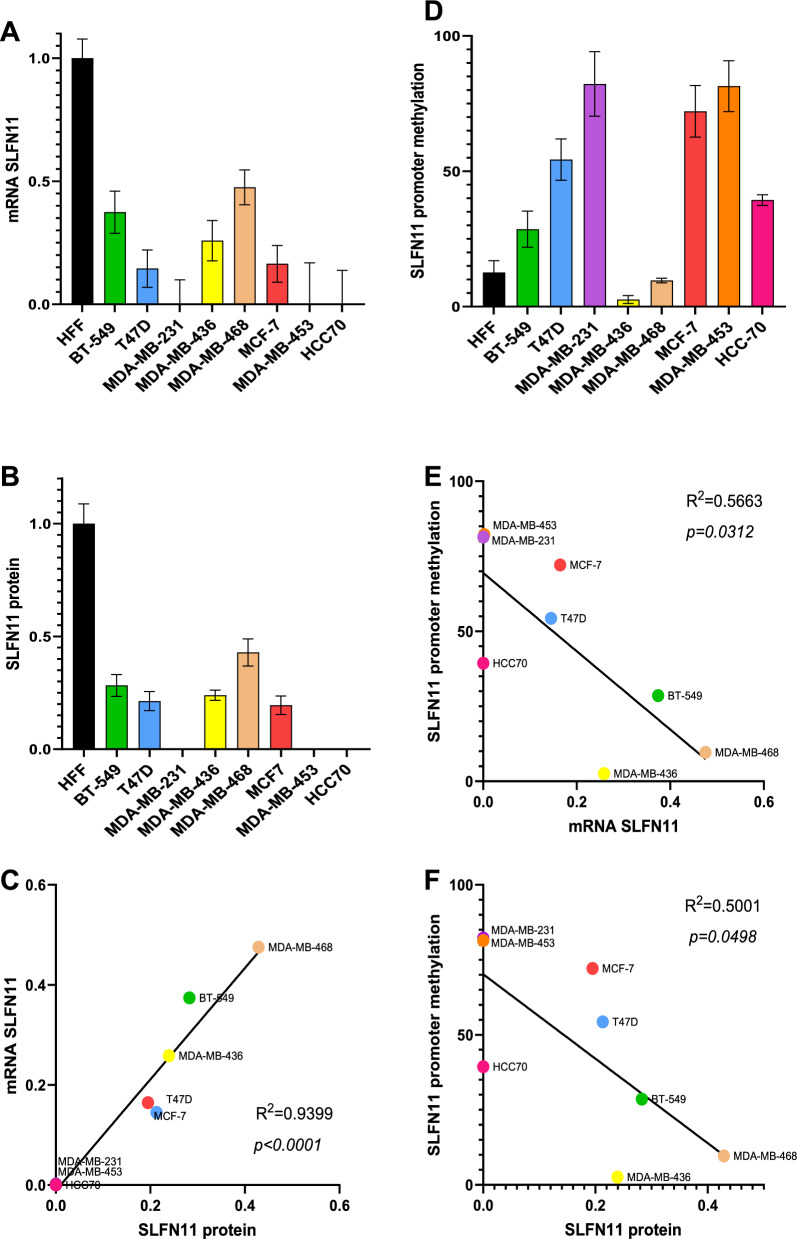
Baseline expression of SLFN11 across a panel of 8 breast cancer cell lines and associated methylation profile. **A** Relative mRNA expression of SLFN11 analyzed by Q-RT-PCR. SLFN11 expression in the different cell lines shown relative to the expression level in HFF (human foreskin fibroblast) used as control cells (N = 6, 3 technical replicates of 2 biological replicates). **B** Capillary Western blot immunoassay (CWB) of SLFN11 expression in those breast cancer cell lines. Quantification of band intensity of SLFN11 relative to the expression level in HFF cells (N = 4, 2 technical replicates of 2 biological replicates). **C** Correlation between SLFN11 mRNA analyzed by Q-RT-PCR and protein expression analyzed by CWB. **D** Percentage of methylation of SLFN11 promoter analyzed by methyl specific PCR (MSP) within the CPG island of the promoter (N = 4, biological replicates). **E** Correlation of SLFN11 promoter methylation and SLFN11 mRNA expression analyzed by Q-RT-PCR. **F** Correlation of SLFN11 promoter methylation and SLFN11 protein expression analyzed by CWB

In order to determine SLFN11 baseline expression in different breast cancer cell lines, BT-549, T47D, MDA-MB-231, MDA-MB-436, MDA-MB-468, MCF-7, MDA-MB-453 and HCC70 were screened for SLFN11 expression both at mRNA and protein level, an immortalized human fibroblasts cell line (HFF) was used as a normal control. We utilized Human Foreskin Fibroblasts (HFF) as a positive control for SLFN11 expression, as previous literature has confirmed the expression of SLFN11 in human primary fibroblasts. The expression in the normal HFF sample was significant and served as a reference point for evaluating the expression in the breast cancer cell lines. However, it's essential to note that this relativity is not used to categorize the expression as normal, low, or high; rather, it is solely employed as a reference for 'normal' cell expression. Analysis by Q-RT-PCR confirms differential SLFN11 mRNA expression among multiple breast cancer cell lines; however, MDA-MB-231, MDA-MB-543 and HCC70 showed almost null SLFN11 mRNA expression (N = 3; Fig. 1A**)**. To validate SLFN11 protein expression, capillary western blot (CWB) was conducted (N = 2; Fig. 1B, Additional file 3: Fig. S3A). In addition, Fig. 1C shows a very significant correlation between mRNA and protein expression of SLFN11 analyzed by Q-RT-PCR and CWB; (R^2^ = 0.86 and p = 0.0008) (Fig. 1C).

Next, to understand what regulates SLFN11 expression, comprehensive methylation analysis of SLFN11 promoter was conducted using methylation specific PCR (MSP) (N = 4; Fig. 1D), a good correlation between promoter methylation and both the mRNA and protein expression was observed (R^2^ = 0.57 and p = 0.029; R^2^ = 0.53 and p = 0.041) (Fig. 1E and 1F). The data shows an increase in SLFN11 methylation, resulting in downregulated mRNA and protein expression of SLFN11,

Therefore, confirming that the methylation of SLFN11 promoter plays a vital role in regulating SLFN11 expression.

### Limited increase in SLFN11 expression upon treatment with Decitabine (DAC)

**Fig. 2:**
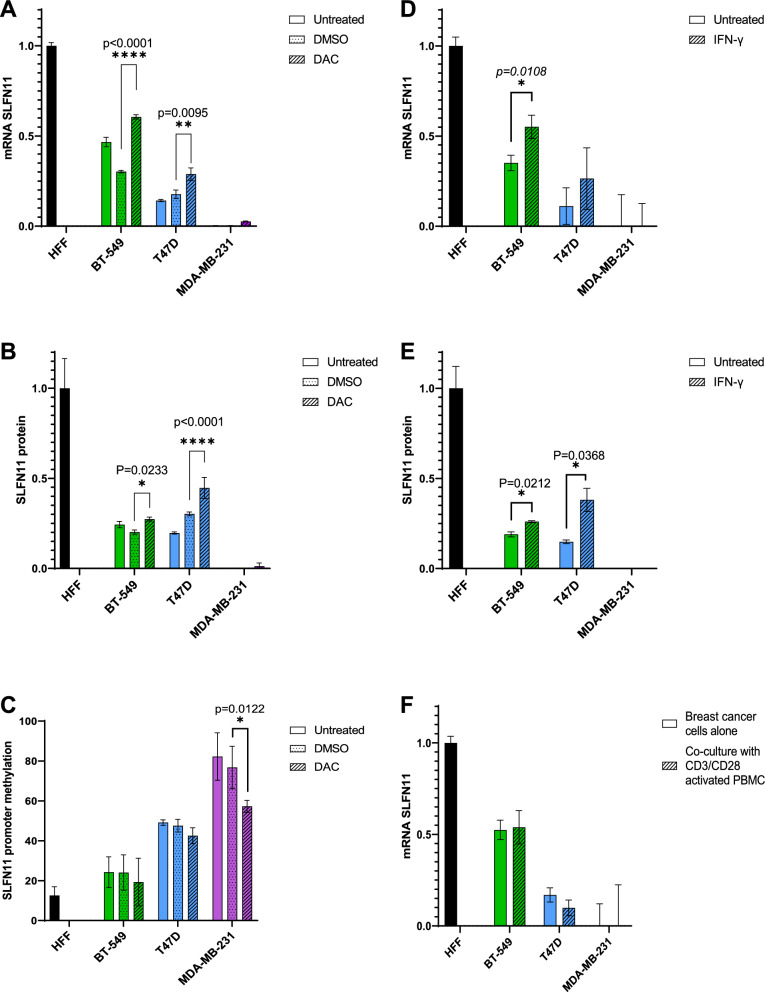
IFN-γ and DAC have limited effect of SLFN11 expression. **A**, **B** Relative expression of SLFN11 analyzed by Q-RT-PCR (N = 3, technical replicates) (**A**) and by CWB (N = 2, technical replicates) (**B**) after treatment with 5 µM of DAC for 72 h compared to the expression level in untreated HFF. **C** Percentage of methylation of SLFN11 promoter analyzed by MSP after treatment with 5 µM of DAC for 72 h (N = 4, biological replicates) compared to untreated HFF. **D**, **E** Relative expression of SLFN11 analyzed by Q-RT-PCR (N = 3, technical replicates) (**D**) and by CWB (N = 2, technical replicates) (**E**) after treatment with 5 nM of IFN-γ for 24 h compared to the expression level in untreated HFF. **F** Relative mRNA expression of SLFN11 analyzed by Q-RT-PCR after co-culture with CD3/CD28 for 24 h compared to the expression level in HFF (N = 3, technical replicates)

Based on the screening results we selected 3 representative breast cancer cell lines (BT-549, T47D and MDA-MB-231) for further experiments as they cover the range of SLFN11 expression observed at baseline (moderate, low and null compared to HFF). In order to re-establish normal SLFN11 expression through demethylation of the SLFN11 gene in the selected breast cancer cell lines, we treated them with 5 µm of DAC for 72 h. We then analyzed SLFN11 mRNA expression using Q-RT-PCR which revealed limited but significant increases in expression in both BT-549 and T47D compared to DMSO treated cells (N = 3; Fig. 2A). Similar increase in the protein expression of SLFN11 in those cells lines was observed was observed (p = 0.0233 for BT-549 and p < 0.0001 for T47D) (N = 2; Fig. 2B, Additional file 3: Fig. S3B). Besides, DAC treatment did not significantly affect the methylation of SLFN11 promoter in BT-549 and T47D breast cancer cell lines. In contrast, the heavily methylated MDA-MB-231 showed almost no effect of DAC on SLFN11 mRNA and protein expression. Even though, there was a significant decrease in SLFN11 promoter methylation in DAC treated MDA-MB-231 cells (p = 0.0122) (N = 4; Fig. 2C).

### Limited increase in SLFN11 expression upon treatment with IFN-γ

Since SLFN11 expression is interferon inducible [26], our next approach was to treat breast cancer cells with 5 nM of IFN-γ for 24 h. Our data shows IFN-γ could increase mRNA expression of SLFN11 in both BT-549 and T47D. However, significance (p = 0.0108) could only be demonstrated in BT-549 cancer cells (N = 3; Fig. 2D). Expression level increases of SLFN11 protein were detected in both BT-549 and T47D (p = 0.0212 and p = 0.0368 respectively) (N = 2; Fig. 2E, Additional file 3: Fig. S3C).

### No increase in SLFN11 expression co-culture with activated PBMC

Finally, we attempted to induce increased SLFN11 expression by co-culturing breast cancer cells with PBMC activated by CD3/CD28 for 24 h. Surprisingly, the results revealed no increment in SLFN11 mRNA expression (Fig. 2F).

### CRISPR-dCas9 can significantly alter SLFN11 expression in BT-549 and T47D cancer cells

Since the increases in SLFN11 expression using DAC or IFN-Ɣ resulted in only minor increases we attempted modulation of SLFN11 expression using UNISAM (Unique Synergistic Activation Mediator) for dead Cas9 (dCas9) activation of SLFN11 [13] and KRAB (Kruppel associated box) for dCas9 inhibition of SLFN11 [27]. We designed 7 gRNAs across the central region of the promoter (Table 2 and Additional file 1: Fig. S1), we then established stable expressing cell lines for all 7 gRNAs. All modified cell lines were screened by CWB and Q-RT-PCR to identify the most efficient single gRNA that could successfully modulate SLFN11 expression levels in the selected cancer cells. The gRNA “N7” was found to be most effective for upregulation of SLFN11 and therefore further used as gRNA for activation and inhibition (Additional file 2: Fig. S2A–I, Additional file 3: Fig. S3 D–G).

**Fig. 3:**
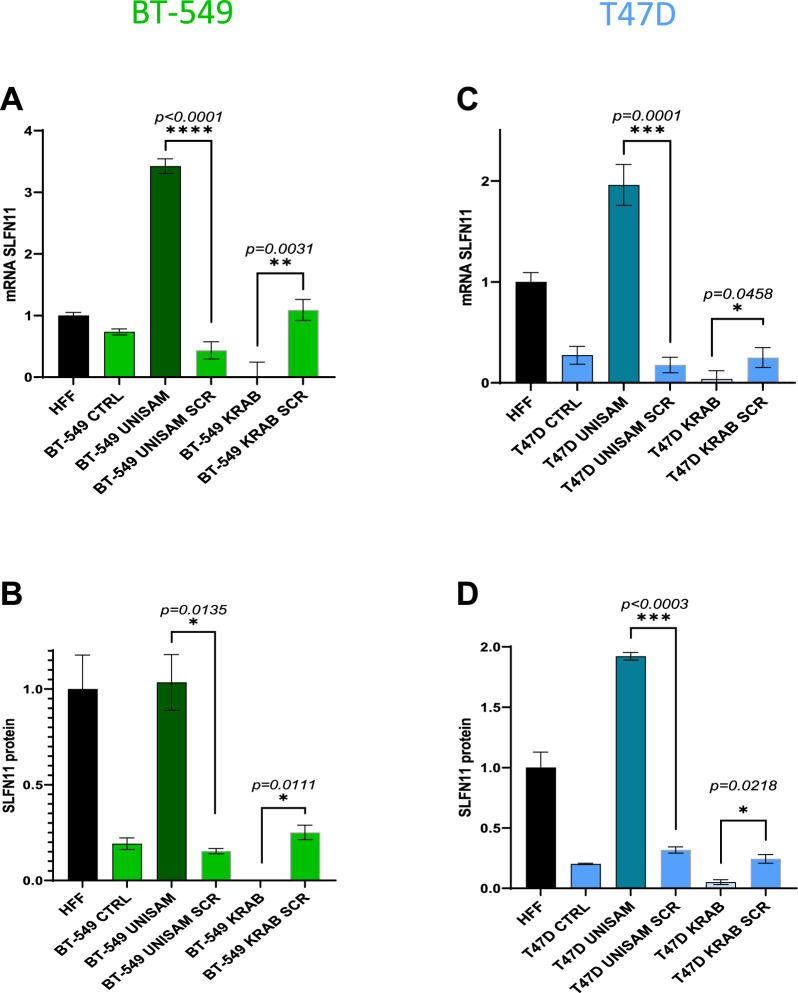
CRISPR-dCas9 system significantly modulate SLFN11 expression. The UNISAM (unique Synergistic Activation Mediator) system developed by Fidanza et al. was used for CRISPR activation of SLFN11 [14] and KRAB (Krüppel associated box) was used for CRISPR inhibition of SLFN11 [14]. **A**, **B** Using the gRNA N7 we could successfully increase (with UNISAM) and decrease (with KRAB) SLFN11 expression in BT-549 as analyzed by Q-RT-PCR (N = 6, 3 technical replicates of 2 biological replicates) (**A**) and CWB (N = 4, 2 technical replicates of 2 biological replicates) (**B**). **C**, **D** Similarly, Using the gRNA N7 we could successfully increase (with UNISAM) and decrease (with KRAB) SLFN11 expression in T47D as analyzed by Q-RT-PCR (N = 6, 3 technical replicates of 2 biological replicates) (**C**) and CWB (N = 4, 2 technical replicates of 2 biological replicates) (**D**). In opposition, the UNISAM and KRAB system used with scramble gRNA (SCR) did not significantly affect SLFN11 expression compared to respective untreated cells (CTRL)

BT-549 and T47D cells were modified with the selected gRNA using UNISAM to increase SLFN11 expression and KRAB to decrease SLFN11 expression. Indeed, SLFN11 mRNA expression is significantly elevated in BT-549 UNISAM (p < 0.0001) and significantly diminished in BT-549 KRAB (p = 0.0031) when compared to respective scramble controls (SCR) (N = 6; Fig. 3A). Likewise, modulation of the SLFN11 protein level in BT-549 cells was significantly higher in UNISAM (p = 0.0135) and lower in KRAB (p = 0.0003) compared to respective scramble controls (N = 4; Fig. 3B, Additional file 3: Fig. S3H). Similarly, T47D cells also showed a significant hike in SLFN11 mRNA expression using UNISAM (p = 0.0001) and a decrease using KRAB (p = 0.0458) in comparison to respective scramble controls (N = 6; Fig. 3C). Also, SLFN11 protein level was significantly increased in UNISAM (p = 0.0003) and decreased in KRAB (p = 0.0215) compared to their respective scrambled controls (N = 4; Fig. 3D**,** Additional file 3: Fig. S3I). However, when working with MDA-MB-231, despite an increase in SLFN11 mRNA expression (Additional file 2: Fig. S2I), no significant amount of protein could be detected in this strongly SLFN11 promoter methylated cell line using UNISAM (Additional file 3: Fig. S3J).

From these data, it is clear that CRISPR-UNISAM and CRISPR-KRAB combined with the appropriate gRNA protospacer (ACACTCGGACAGAATCCTGG) can successfully increase and decrease endogenous SLFN11 expression in BT-549 and T47D cells. This system can modulate SLFN11 expression and is apparently the first report to demonstrate a stable system without inducing cell death.

### Modulation of SLFN11 expression sensitize the cells to Cisplatin, Epirubicin and Olaparib

**Fig. 4:**
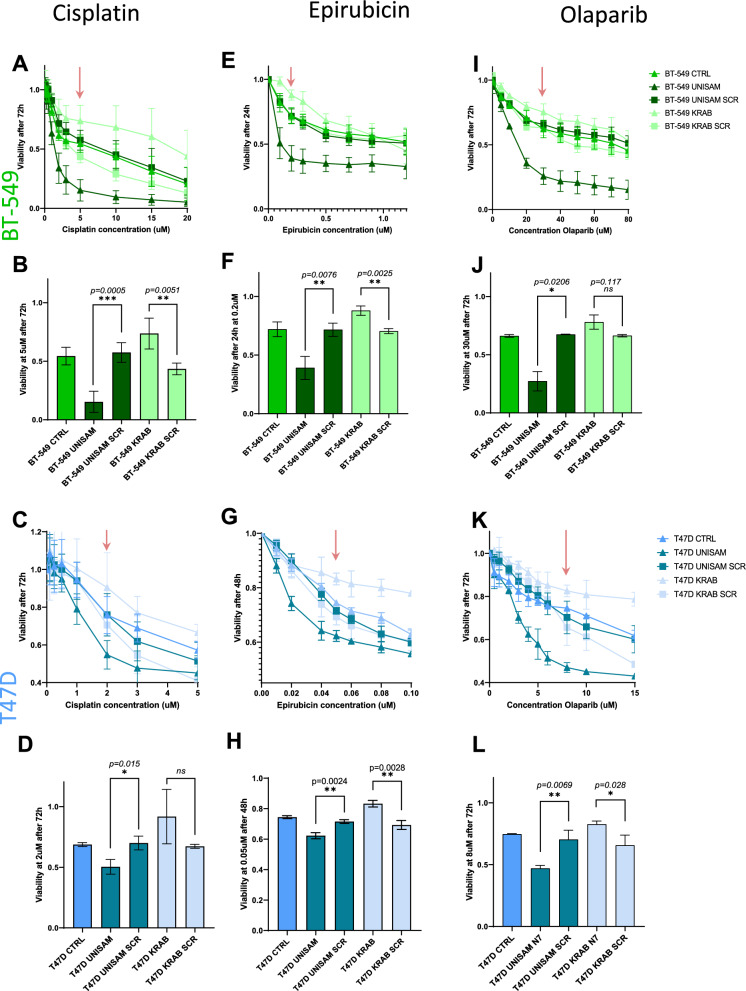
CRISPR-dCas9 modulation of SLFN11 impact sensitivity to DNA Damaging agents. **A**, **B**, **E**, **F**, **I**, **J** In BT-549, SLFN11 increase with UNISAM (gRNA N7) or decrease with KRAB (gRNA N7) leads to respectively significant increase and decreased sensitivity to Cisplatin treatment (N = 4, biological replicates) (**A**, **B**) Epirubicin treatment (**E**, **F**) (N = 4, biological replicates) and Olaparib (N = 4, biological replicates) (**I**, **J**) compared to respective scramble (SCR) controls. Only in Olapraib the decrease of SLFN11 with KRAB (gRNA N7) did not bring significant difference with SCR control at the indicated concentration. **C**, **D**, **G**, **H**, **K**, **L** In T47D, SLFN11 increase with UNISAM (gRNA N7) or decrease with KRAB (gRNA N7) leads to respectively significant increase and decreased sensitivity to Cisplatin treatment (N = 4, biological replicates) (**C**, **D**) Epirubicin treatment (**G**, **H**) (N = 3, biological replicates) and Olaparib (N = 4, biological replicates) compared to respective scramble (SCR) controls. **K**, **L** Only in Cisplatin the decrease of SLFN11 with KRAB (gRNA N7) did not bring significant difference with SCR control at the indicated concentration

The CRIPSR modified cells were then treated with different agents to assess the effect of SLFN11 expression on the sensitivity of cells to chemotherapeutic treatment. For each drugs treatment, different concentration ranges and timepoints were tested, depending on the drug at hand. For each dose response curves we assessed statistical significance at the concentration where nearly maximum effect was observed in the most sensitive cell line before toxicity plateaued (Fig. 4B, D, F, H, J, L).

Cisplatin, one of the most potent and widely used platinum-based drugs, was used to treat our modified BT-549 cells with a concentration ranging from 0.1 µM to 20 µM for 72 h. After treatment cell viability was measured using ATP Lite (N = 3) and Calcein AM (N = 1) (N = 4; Fig. 4A) and expressed relative to the viability of untreated cells (N = 4)**.** We observed that UNISAM modified cells, using the optimal gRNA (UNISAM), showed more sensitivity to cisplatin after 72 h across a wide range of cisplatin concentrations compared to UNISAM using a scrambled gRNA (UNISAM SCR). In opposite, KRAB combined with this gRNA (KRAB) modified cells showed reduced sensitivity to cisplatin after 72 h across a range of cisplatin concentration compared to KRAB used with scrambled gRNA (KRAB SCR). Indeed, at 5 µM, BT-549 UNISAM exhibits significant increased sensitivity compared to UNISAM SCR (p = 0.0005), while BT-549 KRAB showed significant decrease sensitivity compared to KRAB SCR (p = 0.0051) (N = 4; Fig. 4B).

Likewise, in modified T47D cells, upon treatment with Cisplatin for 72 h we observed similar effect, with increased sensitivity of UNISAM modified cells and reduced sensitivity of KRAB modified cells across a range of concentration (N = 4; Fig. 4C). For example, at 2 µM for 72 h, we can see a significant effect on drug sensitivity of T47D UNISAM cells compared to UNISAM SCR (p = 0.015). On the other hand, T47D KRAB cells showed increased viability compared to scrambled, although not reaching significance (N = 4; Fig. 4D**)**.

Epirubicin, belongs to the anthracycline family of chemotherapeutic drugs and is also commonly used for cancer treatment. We treated BT-549 cells with concentrations varying from 0.1 µM to 1.2 µM for 24 h, and again observed increased sensitivity of UNISAM modified cells and reduced sensitivity of KRAB modified cells compared to respective controls (N = 4; Fig. 4E). For example, at 0.2 µM Epirubicin for 24 h BT-549 UNISAM showed significant increase in sensitivity (p = 0.0076) compared to UNISAM SCR, whereas BT-549 KRAB showed decrease sensitivity (p = 0.0025) compared to KRAB SCR (N = 4; Fig. 4F).

T47D cells were also treated with Epirubicin with concentrations varying from 0.01 µM to 0.1 µM for 48 h, and again we observed increased sensitivity of UNISAM modified cells and reduced sensitivity of KRAB modified cells across a range of concentrations (N = 3; Fig. 4G**).** For example, at 0.05 µM, T47D UNISAM showed significant increase in sensitivity (p = 0.0024) compared to UNISAM SCR, whereas BT-549 KRAB showed decrease sensitivity compared to KRAB SCR (p = 0.0028) (N = 3; Fig. 4H).

Olaparib is a Poly (adenosine diphosphate-ribose) polymerase inhibitor (PARPi) and is regarded as a promising anticancer agent. BT-549 cells were treated with Olaparib concentrations varying from 5 µM to 80 µM for 72 h (N = 4; Fig. 4I). Once more, we observed increased sensitivity of UNISAM modified cells and reduced sensitivity of KRAB modified cells in a range of concentration. For instance, at 30 µM, BT-549 UNISAM shows increased sensitivity (p = 0.0206) and BT-549 KRAB showed decreased sensitivity but not significantly compared to relevant scramble controls (N = 4; Fig. 4J). Similarly, T47D cells were treated with Olaparib at concentrations ranging from 0.5 µM to 15 µM for 72 h (N = 4; Fig. 4K). Once more, UNISAM modified cells displayed increased sensitivity KRAB modified cells showed reduced sensitivity compared to their controls. Like at 8 µM, and compared to respective scrambled controls, T47D UNISAM displayed increased sensitivity (p = 0.0069), while T47D KRAB showed decreased sensitivity (p = 0.028) (N = 4; Fig. 4L).

In conclusion, CRISPR-UNISAM and CRISPR-KRAB systems used with appropriate gRNA efficiently increase and decrease SLFN11 expression in BT-549 and T47D which in turn modulates the sensitivity to Cisplatin, Epirubicin, and Olaparib. We can infer that modulation of SLFN11 expression can effectively impact the effect of DNA damaging agents on these cell lines.

### RNAseq analysis of CRISPR modified cells

To further comprehend the mechanism of increased resistance and sensitivity of the CRISPR modified cell lines to cisplatin treatment, we performed RNA sequencing of our modified cells prior and after Cisplatin treatment. Our RNA sequencing was done in triplicate on three independent batches of cells resulting in 5–9 replicates for each condition. The batch effects observed resulting from cell culture and RNA isolation was identified by PCA (principal component analysis) analysis (Additional file 4: Fig. S4). This batch effect was then resolved using Combat [19].

**Fig. 5:**
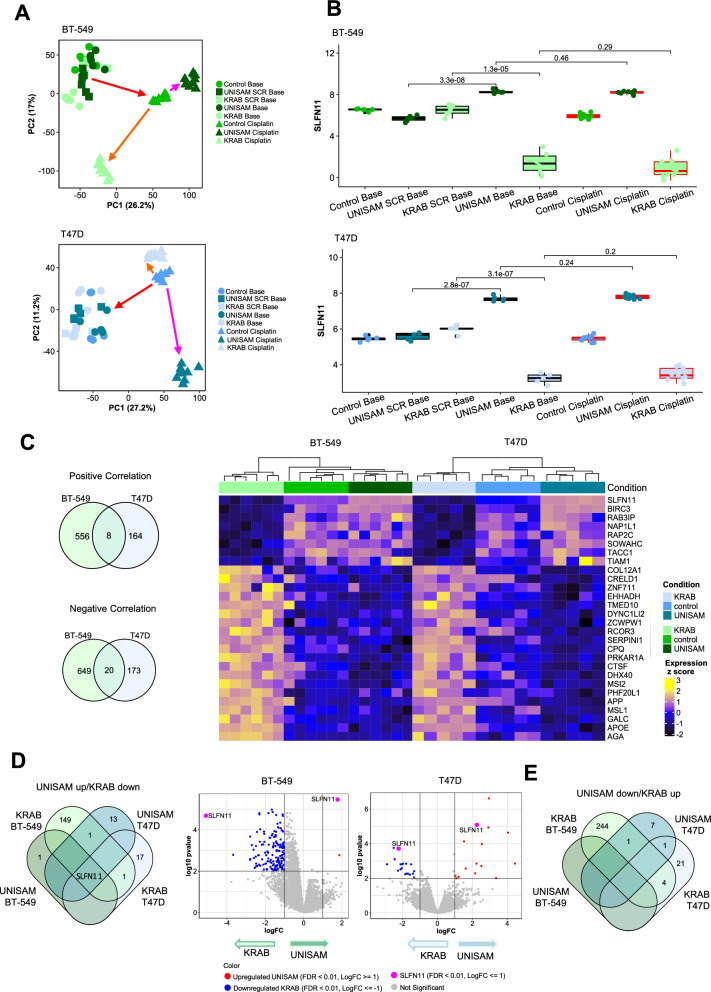
RNAseq analysis of CRISPR modified cells. **A** Principal component analysis based on gene expression for all samples (baseline and cisplatin-treated samples) post performing combat on samples to remove batch effect for BT-549 and T47D cell lines. **B** Expression of SLFN11 across baseline and cisplatin-treated samples in BT-549 and T47D cell lines. Statistical significance was assessed using an unpaired t test. **C** VennDiagram of common genes between BT-549 and T47D which are positively (N = 8) and negatively (N = 20) correlating with SLFN11 (correlation coefficient R <|0.5|) based on the baseline samples. Heatmap showing the expression of positively and negatively correlating genes with SLFN11 (N = 28). **D** VennDiagram of differentially expressed genes between BT-549 and T47D cell lines in UNISAM up (vs. control) and KRAB down (vs. control) (FDR < 0.01, logFC <|1|). Volcano plot showing upregulated DEG between UNISAM vs, control and downregulated DEG between KRAB vs, control in baseline samples (FDR < 0.01, logFC <|1| considered as significant). **E** VennDiagram of differentially expressed genes between BT-549 and T47D cell lines in UNISAM down (vs. control) and KRAB up (vs. control) (FDR < 0.01, logFC <|1|) in baseline samples. BT-549 CTRL and modified cells (UNISAM and KRAB) N = 6. T47D CTRL and modified cells (UNISAM and KRAB) N = 5. Cisplatin treated samples for each cell line N = 9 for all analysis performed

PCA analysis clearly shows a separation between the Cisplatin treated and non-treated cells. Indeed, while the CRISPR modified cells clustered close to unmodified or scramble modified cells, the Cisplatin treatment of cells resulted in a drastic shift of the treated cells (represented by the red arrows in Fig. 5A). Under Cisplatin treatment the effect of the CRISPR modification between UNISAM (shift indicated in pink), KRAB (shift indicated in orange) and unmodified cells is captured by a different dimension in the PCA plot.

In alignment with our Q-RT-PCR and western blot analysis, RNA sequencing confirmed that UNISAM modification of both BT-549 and T47D resulted in a strong increase of SLFN11 RNA expression in baseline (p = 3,3.10^–8^ and p = 2,8.10^–7^ respectively), while KRAB significantly reduced the expression of SLFN11 in baseline (p = 1,3.10^–5^ and p = 3,1.10^–7^ respectively). Of note, Cisplatin treatment did not affect SLFN11 expression in UNISAM in BT-549 and T47D (p = 0.46 and p = 0.24 respectively) as well in KRAB (p = 0.29 and p = 0.2 respectively) when compared to corresponding baseline samples (Fig. 5B).

To appreciate the consistency of the perturbations caused by SLFN11 modulation across cell lines we performed Pearson correlation and analyzed the genes that were positively or negatively correlated (R > +/− 0.5) with SLFN11 expression in both UNISAM and KARB modified BT-549 and T47D cell lines. Among those genes 8 were positively, and 20 were negatively correlated with SLFN11 in both cell lines (Fig. 5C).

The analysis of differentially expressed genes (DEGs) using a log fold change ≥ 1 and FDR ≤ 0.01, showed that only a few genes were upregulated or down regulated along with SLFN11 in UNISAM and KRAB in each cell lines compared to controls modified with a scrambled guide RNA (volcano plots in Fig. 5D). Though, only SLFN11 was commonly up regulated in UNISAM and downregulated in KRAB between the two cell lines (Fig. 5D). No common genes between the two cell lines could be identified as down regulated in UNISAM and upregulated in KRAB (Fig. 5E). This data confirms the specificity of the UNISAM and KRAB CRISPR systems for up or down regulating SLFN11 specifically.

### RNASeq analysis of CRISPR modified cells treated with Cisplatin

**Fig. 6:**
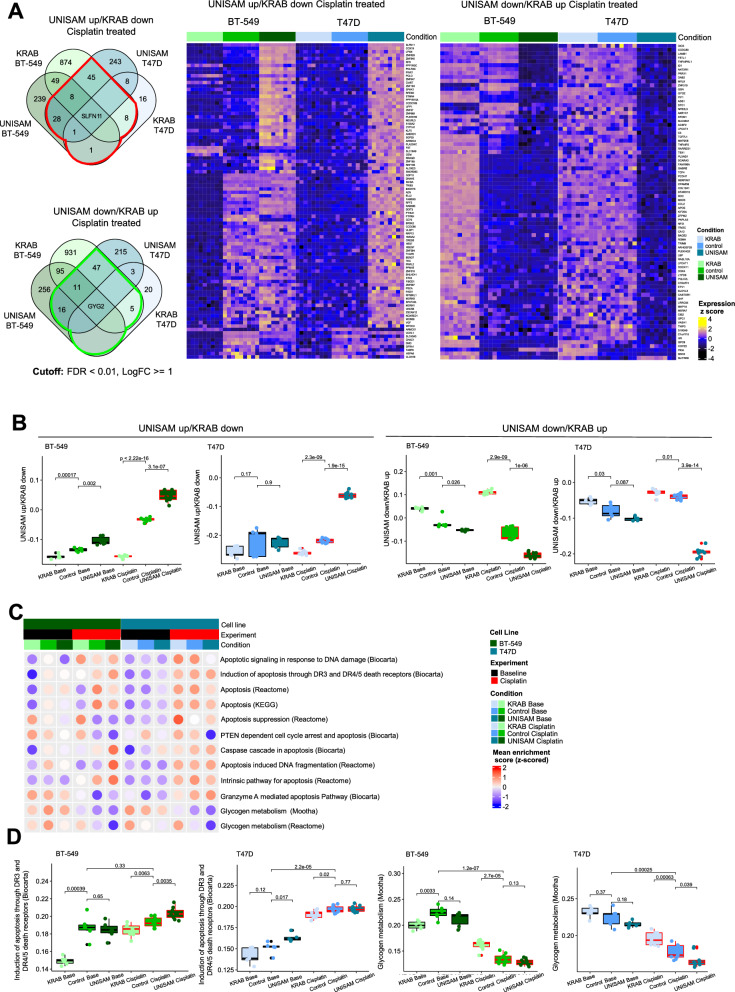
RNASeq analysis of CRISPR modified cells treated with Cisplatin. **A** VennDiagram of differentially expressed genes between BT-549 and T47D cell lines in UNISAM up/KRAB down (vs. control), and UNISAM down/KRAB up (vs. control) respectively, (FDR < 0.01, logFC <|1|) in Cisplatin-treated samples. Heatmaps illustrate the expression of DEG genes in UNISAM up/KRAB down and UNISAM down/KRAB up (genes highlighted in red and green in VennDiagram, respectively). **B** Boxplot of enrichment score generated for list of DEG (UNISAM up/KRAB down (n = 94) and UNISAM down/KRAB up (n = 87)) in BT-549 and T47D cell lines. Statistical significance was assessed using an unpaired t test (**C**). Dotted heatmap of mean enrichment score for apoptosis and glycogen metabolism pathways. **D** Boxplot of enrichment scores for selected apoptosis and glycogen metabolism pathways in BT-549 and T47D cell lines Statistical significance was assessed using an unpaired t test. BT-549 CTRL and modified cells (UNISAM and KRAB) N = 6. T47D CTRL and modified cells (UNISAM and KRAB) N = 5. Cisplatin treated samples for each cell line N = 9 for all analysis performed

To identify genes associated with SLFN11 up and down-regulation under Cisplatin treatment in both cell lines, we performed DEG analysis comparing UNISAM and KRAB modified cells with unmodified controls, using Limma and considered genes with log fold change ≥ 1 and FDR ≤ 0.01 as significant. A list of DEGs comparing cells modified with scrambled gRNA’s vs unmodified cells was used to remove false positives. When only considering genes affected in the same way in both cell lines there are 92 genes upregulated in UNISAM/downregulated in KRAB under cisplatin treatment (red). On the other hand, 80 genes were found to be downregulated in UNISAM/upregulated in KRAB under cisplatin treatment (green) (Fig. 6A).

In order to reduce the dimensions of the DEG matrix an enrichment score (ES) of genes upregulated in UNISAM/downregulated in KRAB (n = 92) and genes downregulated in UNISAM/upregulated in KRAB (n = 80) was calculated using ssGSEA to create a SLFN11 signature for genes that go in the same or opposite direction as SLFN11, respectively. As expected under cisplatin treatment this upregulated in UNISAM/downregulated in KRAB score is low in KRAB, high in UNISAM with the control cells in the middle and the reverse seen in the when looking at the downregulated in UNISAM/upregulated in KRAB score. What is interesting is that the same pattern, although much weaker, is observed in the cell not treated with cisplatin, indicating the change in DNA Damage response machinery (Fig. 6B).

Next, we aimed to identify the enriched pathways using genes from the DEG analysis. The above-mentioned gene lists were uploaded to Ingenuity Pathway Analysis (IPA) database. Results show that there are 22 pathways that show enrichment of genes in these gene lists (Additional file 5: Fig. S5). However, we could not identify pathways associated with cell death and apoptosis as one would expect. This incentivized us to perform a more guided pathway analysis by looking specifically at apoptosis pathways, in addition to glycogen metabolic pathways since we found that GYG2 was consistently downregulated in UNISAM and upregulated in KRAB in both BT-549 and T47D cell lines treated with Cisplatin. To do so, we downloaded gene sets belonging to these pathways from Molecular Signatures Database (MSigDB) and calculated an ES for each pathway and visualized these in a heatmap (Fig. 6C). As expected, enrichment scores show that most apoptosis pathways have higher enrichment in Cisplatin treated samples compared to baseline, and the reverse is observed for glycogen metabolism pathways. To illustrate the effect SLFN11 has in this context, we selected the pathways showing the clearest trend in both cell lines (Fig. 6D). Obviously, higher enrichment of apoptosis pathway is observed in Cisplatin treated samples compared to control. More interestingly, within Cisplatin treated sample, apoptosis is significantly more enriched in UNISAM and less enriched in KRAB in BT-549 (p = 0.0035, p = 0.0063 respectively), however in T47D enrichment was only significantly higher in KRAB (p = 0.02) when compared to corresponding controls. Whereas for glycogen metabolism pathway, lower enrichment was observed in Cisplatin treated samples compared to baseline samples. Glycogen metabolism is significantly higher enriched in KRAB in BT-549 and T47D (p = 2,7.10^–5^, p = 0.00063 respectively), whereas it was significantly less enriched in UNISAM in T47D only (p = 0.039) compared to control. This would be in line with the higher energetic requirements of a proliferating cell or a cell with active DNA repair machinery [28].

## Discussion

SLFN11 was identified in 2012 as a prognostic marker for response to DNA damaging agents in-silico [6]) and was subsequently found to affect treatment response in brain and prostate cancer cells through its role in replication checkpoint maintenance and homologous recombination repair [1]. This is further substantiated by the negative correlation of SLFN11 immunohistochemical staining with cisplatin treatment response in ovarian cancer [7]. It was therefore postulated that the opposite should also be true. Here, we utilized two different approaches to investigate the effect of manipulating SLFN11 expression on tumor cell viability and treatment response to DNA damaging agents. Traditional overexpression of SLFN11 induced significant cell death and compromised cellular fitness to intolerable levels (data not shown). Since SLFN11 expression has been shown to be inducible by IFN [11], and regulated by methylation, we explored both mechanisms to increase endogenous SLFN11 expression in cancer cell lines [3, 29]. Using the demethylation agent DAC and ectopic IFN- γ we were able to induce a moderate increase in SLFN11 expression in T47D and BT-549 cell line. Though, this increase, despite being statistically significant, was limited. We therefore attempted a more specific increase in SLFN11 expression using a dCas9 CRISPR activation system (UNISAM), we obtained a strong induction of SLFN11 expression, resulting in increased sensitivity to DNA damaging agents and Topo isomerase inhibition. Conversely, eSLFN11 downregulation using a dCas9 CRISPR inhibition system (KRAB) reduced treatment response similar treatments in the same cell lines. By transcriptomic analysis we could establish the specificity of those CRISPR systems used to up and down regulate SLFN11 as the only gene upregulated by the UNISAM system in both T47D and BT-549 was SLFN11.

Of note, changes in treatment responses were only observed in cell lines with residual SLFN11 expression and moderate methylation of its promoter (50% methylation in the case of T47D). Cell lines that completely lack SLFN11 expression due to strong promoter hypermethylation such as MDA-MB-231 breast cancer cell lines, did not exhibit an increase in SLFN11 expression upon dCas9 CRISPR activation. This observation tends to demonstrate that although introducing transcription factors, such as those integrated into the SAM system, can promote increased transcription and subsequent SLFN11 production, it may fall short when faced with more robust down-regulation imposed by hypermethylation. Interestingly, treatment with a general demethylating agent like DAC did lead to a partial demethylation of the SLFN11 promoter; however, it too proved inadequate in restoring significant protein expression. While increasing SLFN11 expression can be beneficial in many clinical cases, it may be the most beneficial in patients without any SLFN11 expression in the tumor. This underscores the considerable challenge associated with treating patients whose tumors exhibit no detectable SLFN11 expression whatsoever. Therefore, we believe that a combination of SLFN11-treatment strategies should be further explored.

We believe in-dept exploration of the feedback loop observed between tumor cells SLFN11 expression and T-cells IFN-γ expression is required to understand the DNA damage response mechanisms interactions with the anti-tumor immune response [7, 26]. Therefore, it is worth testing in-vivo if the combined treatment with previously approved drugs like demethylation agents such as DAC combined with immune checkpoint blockade could lead to sufficient increase in SLFN11 expression in tumors with a decreased SLFN11 expression. Additionally, it's crucial to note that the scope of this work is restricted to breast cancer cell lines. It is important to validate these findings in other prominent cancer cell types where the correlation between SLFN11 expression and chemosensitivity has been established, such as lung, colon or prostate cancer. Another critical aspect to consider is the possibility of intra-tumoral heterogeneity, as evidenced by studies [30, 31]. This heterogeneity presents a significant challenge for effective treatment strategies but also offers an opportunity to deepen our understanding of the biological role of SLFN11 [32]. In cases where tumors exhibit heterogeneity in SLFN11 expression, chemotherapy treatment may be more effective against specific intra-tumoral clones with higher SLFN11 expression, potentially leading to chemoresistanceSLFN11 [33]. This highlights the importance of employing multiregional sequencing techniques [34]. Furthermore, assessing the SLFN11 status in relapsed tumors compared to primary tumors may give us more insight into these scenarios.

If using these existing agents would not meaningfully improve chemosensitivity, more potent approaches like an in-vivo application of the dCAS9 method could be evaluated. Also, while clinical phase I trials using CRISPR are on the rise, the use of CRISPR systems such as activation system require permanent expression in target cell and could lead to additional adverse effect due to integration of the constructs. Alternatively, other dCAS CRISPR systems allow specific demethylation of chosen genes promoters. As methylation is key to unlocking SLFN11 expression, it would be worth investigating if in such resistant cell lines, CRISPR driven specific demethylation would be sufficient to stably increase SLFN11 expression alone or in combination with other treatments.

Moreover, it is worth noting that as human clinical trials utilizing CRISPR technology are currently underway, concerns regarding the safety of CRISPR technology in clinical and translational applications have become the subject of intense debate [35, 36].

## Conclusions

Increase in SLFN11 expression was achieved with IFN-γ, DAC demethylation and a dCAS CRISPR activation system, leading to increased sensitivity to DNA damage repair pathway related drugs. In-vivo testing with existing agents or in combination with immune checkpoint blockade could lead to sufficient SLFN11 expression to modulate chemosensitivity. Further understanding of the feedback loop between tumor cells SLFN11 expression and T-cells IFN-γ expression is required. Alternatively, specific demethylation using other novel dCAS CRISPR systems could increase SLFN11 expression in resistant cell lines.

### Supplementary Information

**Additional file 1:** gRNA location and CRISPR modification. (**Fig. S1A**) The predicted promoter region of SLFN11 (in green) is surrounding the gene’s exon1 and CpG island (in red) analysis show its location in the center of the promoter area. gRNAs were therefore designed along the central region of the promoter of SLFN11 (N1 to N10). (**Fig. S1B**) Schematic representation of the strategy adopted to respectively increase SLFN11 expression using UNISAM system and decrease SLFN11 expression using KRAB system. After insertion of the gRNA into the respective plasmids, cells were transformed with the integrative plasmids using electroporation and selected for the expression of respectively mCherry or GFP reporter genes. Cells were then analyzed for SLFN11 expression by westernblot and by Q-RT-PCR.
**Additional file 2:** Screening of gRNA efficiency at upregulating or downregulating SLFN11 using UNISAM or KRAB systems. (**Fig. S2A**–**Fig. S2D**) Relative mRNA expression of SLFN11 analyzed by Q-RT-PCR (N = 3, technical replicates) (**Fig. S2A**–**Fig. S2C**) and relative SLFN11 protein expression analyzed by CWB (N = 2, technical replicates) (**Fig. S2B**–**Fig. S2D**) in BT-549 cancer cell lines modified with each gRNA for CRISPR-dCas9-UNISAM (**Fig. S2A**, **Fig. S2B**) or CRISPR-dCas9-KRAB (**Fig. S2C**, **Fig. S2D**) relative to non-modified cell line. (**Fig. S2E**–**Fig. S2H**) Relative mRNA expression of SLFN11 analyzed by Q-RT-PCR (N = 3, technical replicates) (**Fig. S2E**–**Fig. S2G**) and relative SLFN11 protein expression analyzed by CWB (N = 2, technical replicates) (**Fig. S2F**–**Fig. S2H**) in T47D cancer cell lines modified with each gRNA for CRISPR-dCas9-UNISAM (**Fig. S2E**–**Fig. S2F**) or 5 (N1, N2, N6, N7 and N10) of the 7 gRNA for CRISPR-dCas9-KRAB (**Fig. S2G**, **Fig. S2H**) relative to non-modified cell line. (**Fig. S2I**) Relative mRNA expression of SLFN11 analyzed by Q-RT-PCR in MDA-MB-231 cancer cell lines modified with each gRNA for CRISPR-dCas9-UNISAM relative to non-modified cell line.
**Additional file 3:** Representative CWB results. (**Fig. S3A**) Representative CWB results of the analysis of SLFN11 protein expression in the 8 tested unmodified breast cancer cell lines compared to HFF. (**Fig. S3B**) Representative SLFN11 protein expression in BT-549, T47D and MDA-MB-231 analyzed by CWB after treatment with 5uM of DAC for 72 h compared to the expression level in untreated HFF. (**Fig. S3C**) Representative SLFN11 protein expression in BT-549, T47D and MDA-MB-231 analyzed by CWB after treatment with 5 nM of IFN-γ for 24 h compared to the expression level in untreated HFF. (**Fig. S3D**–**Fig. S3E**) Representative SLFN11 protein expression in BT-549 (D) or T47D (**Fig. S3E**) modified with UNISAM and each of the 7 gRNA compared to the respective unmodified cells. (**Fig. S3F**-**Fig. S3G**) Representative SLFN11 protein expression in BT-549 (**Fig. S3F**) or T47D (**Fig. S3G**) modified with KRAB and each of the 7 gRNA compared to the respective unmodified cells. (**Fig. S3H**-**Fig. S3I**) Representative SLFN11 protein expression level analyzed by CWB in BT-549 (**Fig. S3H**) or T47D (**Fig. S3I**) after modification with UNISAM (gRNA 7 or gRNA SCR) or KRAB (gRNA 7 or gRNA SCR) compared to respective unmodified cells and HFF. (**Fig. S3J**) Representative SLFN11 protein expression level analyzed by CWB in MDA-MB-231 after modification with UNISAM (gRNA 7) compared to respective unmodified cells and HFF.
**Additional file 4: Fig. S4.** Principal component analysis pre and post-combat. Principal component analysis based on gene expression for all samples (baseline and cisplatin-treated samples) pre and post performing combat on samples to remove batch effect for BT-549 and T47D cell lines.
**Additional file 5: Fig. S5** Pathway enrichment analysis. Enriched pathways associated with DEG (n = 181, FDR < 0.01, LogFC >  = 1) from limma analysis in UNISAM up/KRAB down and UNISAM down/KRAB up, using Ingenuity Pathway Analysis (IPA).

## Data Availability

The datasets generated and/or analyzed during the current study are available in the FigShare repository, https://doi.org/10.6084/m9.figshare.22776191.

## Abbreviations

SLFN11: Schlafen family member 11
ATR: ataxia-telangiectasia-mutated-and-Rad3-related kinase
CHK 1: checkpoint kinase 1
RPA: Replication Protein A
MCM3: Minichromosomal maintenance complex component 3
DHX9: DExH-box helicase
DDAs: DNA Damaging Agents
DDRs: DNA Damage Response
PARPi: Poly (adenosine diphosphate-ribose) polymerase inhibitor
PRC: Polycomb Repressive Complex
IFN-γ: Interferon gamma
DAC: Decitabine
PBMCs: Peripheral blood mononuclear cell
RPMI: Roswell Park Memorial Institute
FBS: Fetal bovine serum
Q-RT-PCR: Quantitative Real time Polymerase Chain Reaction
PCR: Polymerase chain reaction
DPBS: Dulbecco's phosphate-buffered saline
CWB: Capillary Western Blot
MSP: Methyl Specific Polymerase Chain Reaction
DMSO: Dimethyl sulfoxide
PBL: Peripheral blood lymphocyte
T:E: Target:Effector
gRNA: Guide RNA
SCR: Scramble
PCA: Principal component analysis
ES: Enrichment Score
DEGs: Differentially expressed genes
ssGSEA: Single sample gene set enrichment analysis
MSigDB: Molecular Signatures Database
IPA: Ingenuity Pathway Analysis
dCas9: Dead CRISPR associated protein 9
UNISAM: Unique Synergistic Activation Mediator
KRAB: Kruppel associated box
UNISAM SCR: UNISAM using a scrambled gRNA
KRAB SCR: KRAB used with scrambled gRNA
gRNA: Guide RiboNucleic acid
sgRNA: Single guide RiboNucleic acid
