# Supplementary material for: Modulation of SLFN11 induces changes in DNA Damage response in breast cancer
Source: Cancer Cell Int. 2023 Nov 24;23:291. doi: 10.1186/s12935-023-03144-w (PMC10668346; doi:10.1186/s12935-023-03144-w)
Supplement: Supplementary file 1 — Additional file 1: gRNA location and CRISPR modification. (Fig. S1A) The predicted promoter region of SLFN11 (in green) is surrounding the gene’s exon1 and CpG island (in red) analysis show its location in the center of the promoter area. gRNAs were therefore designed along the central region of the promoter of SLFN11 (N1 to N10). (Fig. S1B) Schematic representation of the strategy adopted to respectively increase SLFN11 expression using UNISAM system and decrease SLFN11 expression using KRAB system. After insertion of the gRNA into the respective plasmids, cells were transformed with the integrative plasmids using electroporation and selected for the expression of respectively mCherry or GFP reporter genes. Cells were then analyzed for SLFN11 expression by westernblot and by Q-RT-PCR. Additional file 2: Screening of gRNA efficiency at upregulating or downregulating SLFN11 using UNISAM or KRAB systems. (Fig. S2A–Fig. S2D) Relative mRNA expression of SLFN11 analyzed by Q-RT-PCR (N = 3, technical replicates) (Fig. S2A–Fig. S2C) and relative SLFN11 protein expression analyzed by CWB (N = 2, technical replicates) (Fig. S2B–Fig. S2D) in BT-549 cancer cell lines modified with each gRNA for CRISPR-dCas9-UNISAM (Fig. S2A, Fig. S2B) or CRISPR-dCas9-KRAB (Fig. S2C, Fig. S2D) relative to non-modified cell line. (Fig. S2E–Fig. S2H) Relative mRNA expression of SLFN11 analyzed by Q-RT-PCR (N = 3, technical replicates) (Fig. S2E–Fig. S2G) and relative SLFN11 protein expression analyzed by CWB (N = 2, technical replicates) (Fig. S2F–Fig. S2H) in T47D cancer cell lines modified with each gRNA for CRISPR-dCas9-UNISAM (Fig. S2E–Fig. S2F) or 5 (N1, N2, N6, N7 and N10) of the 7 gRNA for CRISPR-dCas9-KRAB (Fig. S2G, Fig. S2H) relative to non-modified cell line. (Fig. S2I) Relative mRNA expression of SLFN11 analyzed by Q-RT-PCR in MDA-MB-231 cancer cell lines modified with each gRNA for CRISPR-dCas9-UNISAM relative to non-modified cell line. Additional file 3: Representative CWB results. [file 12935_2023_3144_MOESM1_ESM.pptx]

## Slide 1
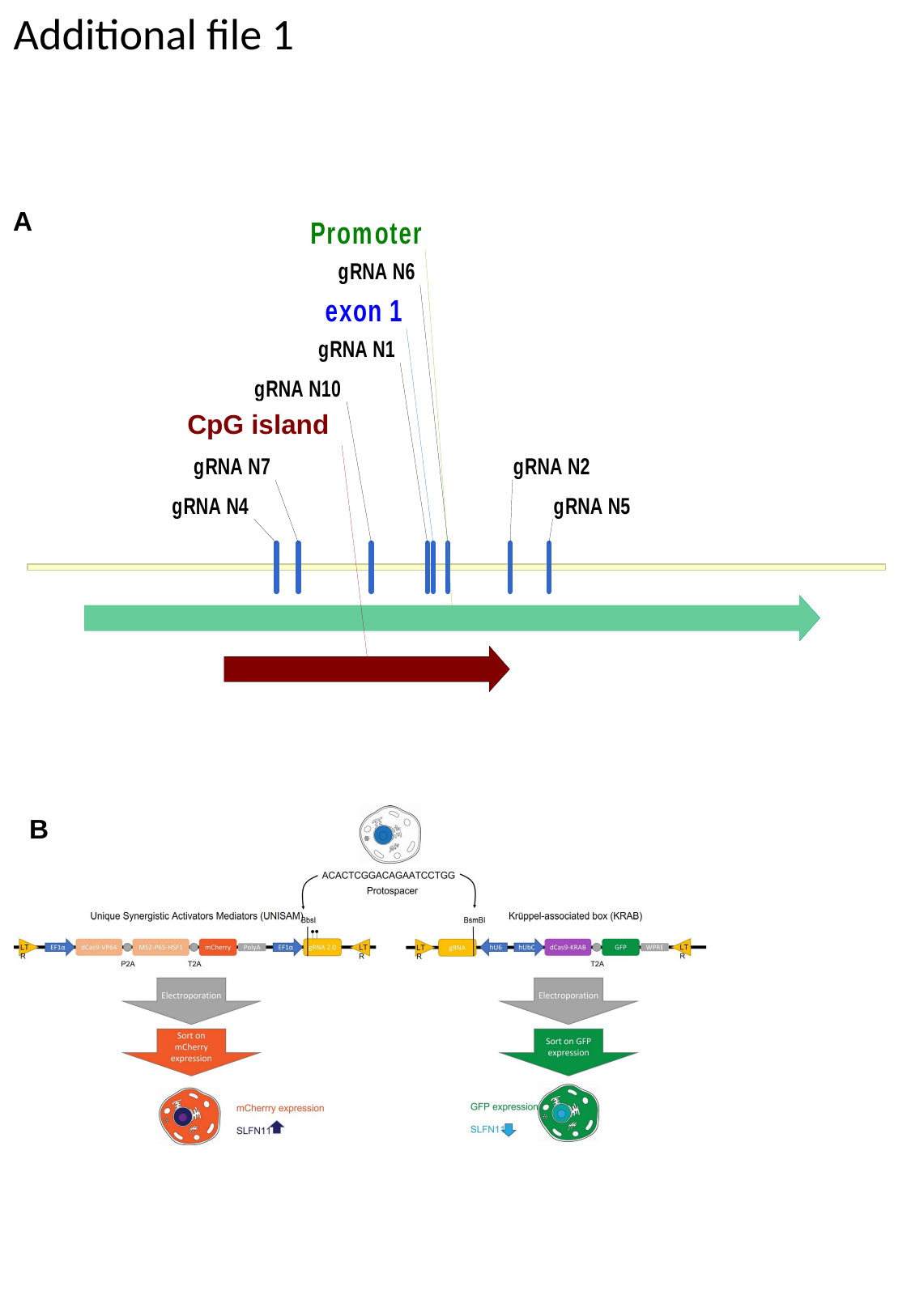

Additional file 1
A
CpG island
B

## Slide 2
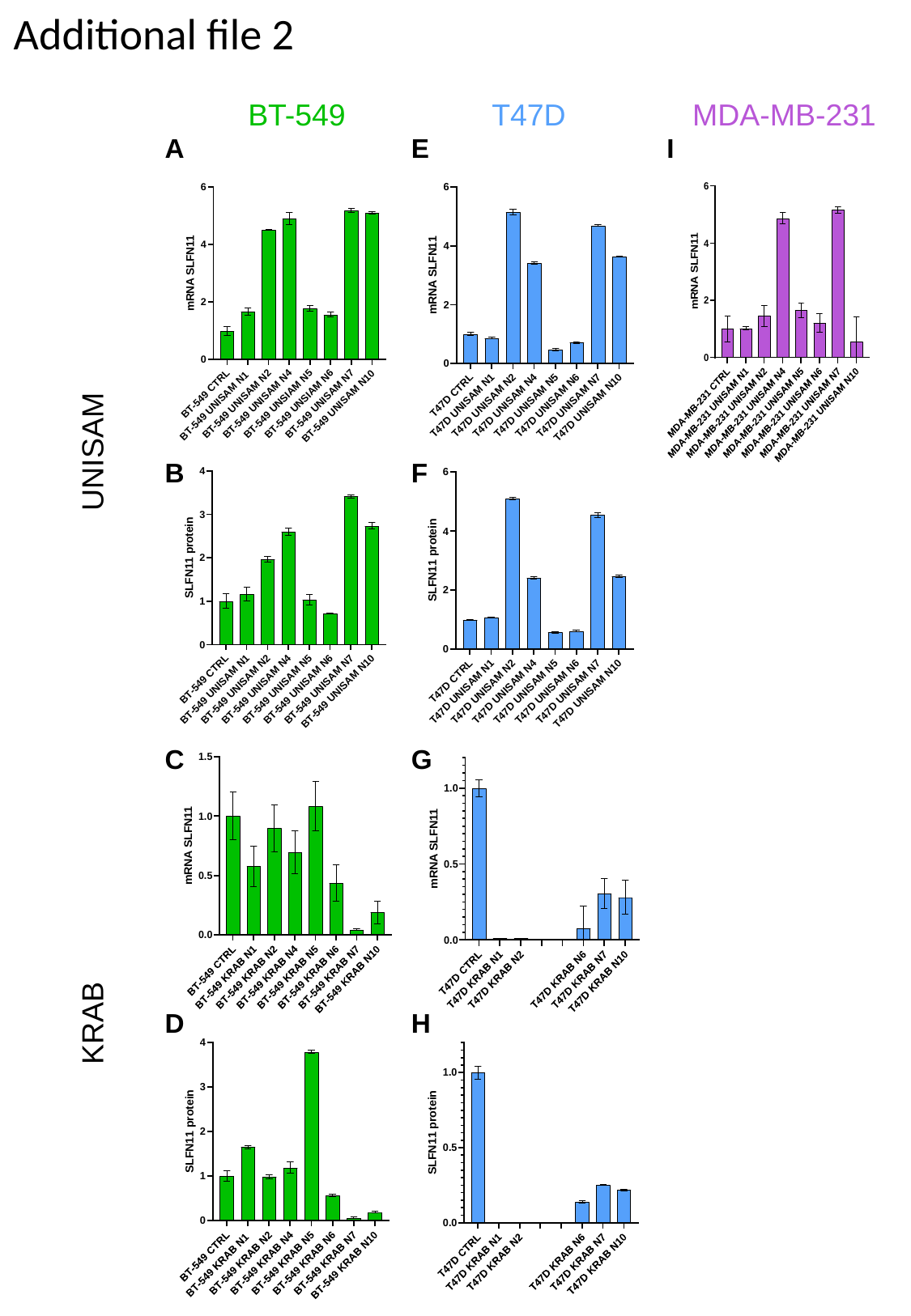

Additional file 2
BT-549
T47D
MDA-MB-231
A
E
I
UNISAM
B
F
C
G
KRAB
D
H

## Slide 3
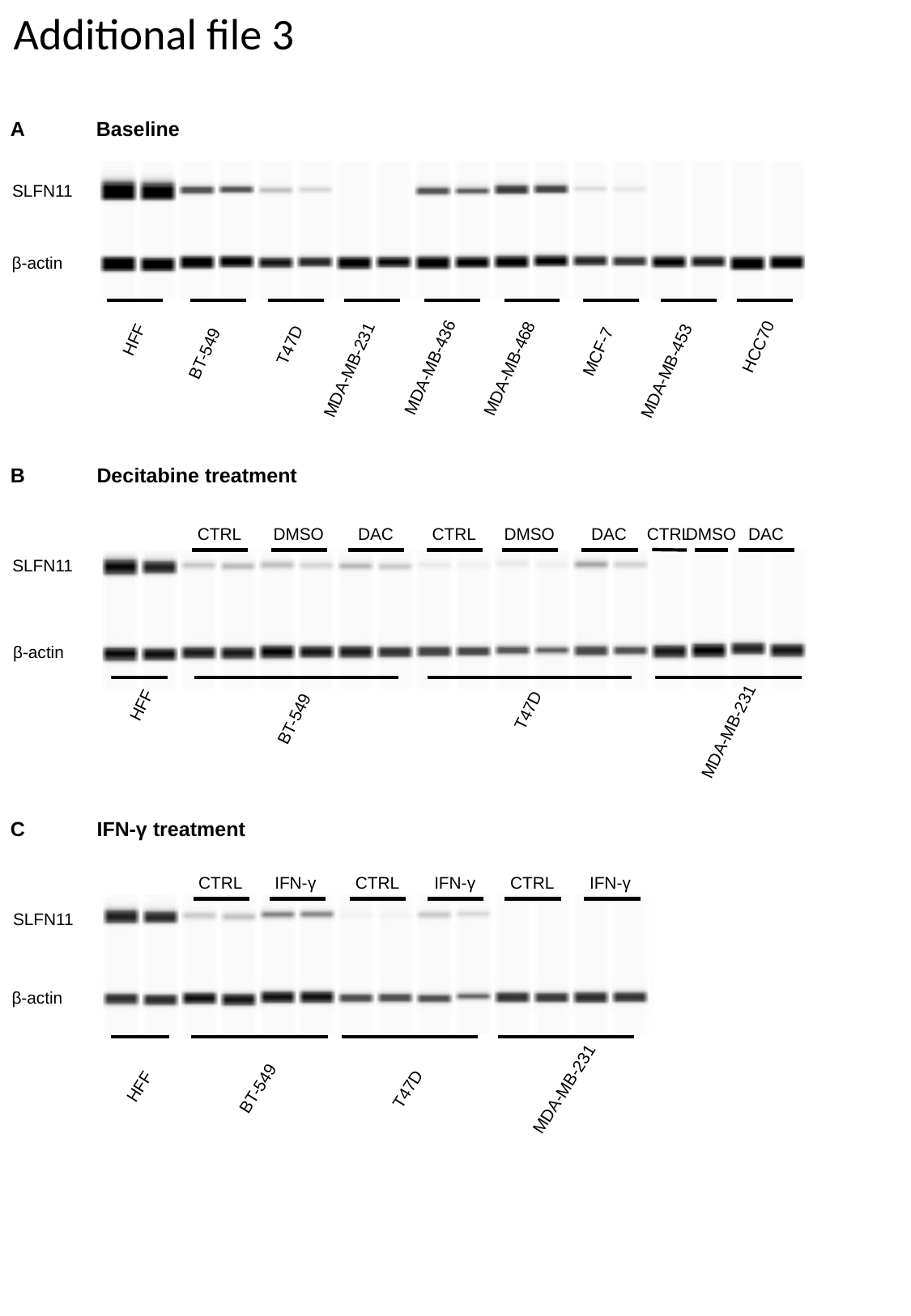

Additional file 3
A
Baseline
SLFN11
β-actin
HFF
T47D
HCC70
BT-549
MCF-7
MDA-MB-436
MDA-MB-468
MDA-MB-231
MDA-MB-453
B
Decitabine treatment
CTRL
DMSO
DAC
CTRL
DMSO
DAC
CTRL
DMSO
DAC
SLFN11
β-actin
HFF
T47D
BT-549
MDA-MB-231
C
IFN-γ treatment
CTRL
IFN-γ
CTRL
IFN-γ
CTRL
IFN-γ
SLFN11
β-actin
HFF
BT-549
T47D
MDA-MB-231

## Slide 4
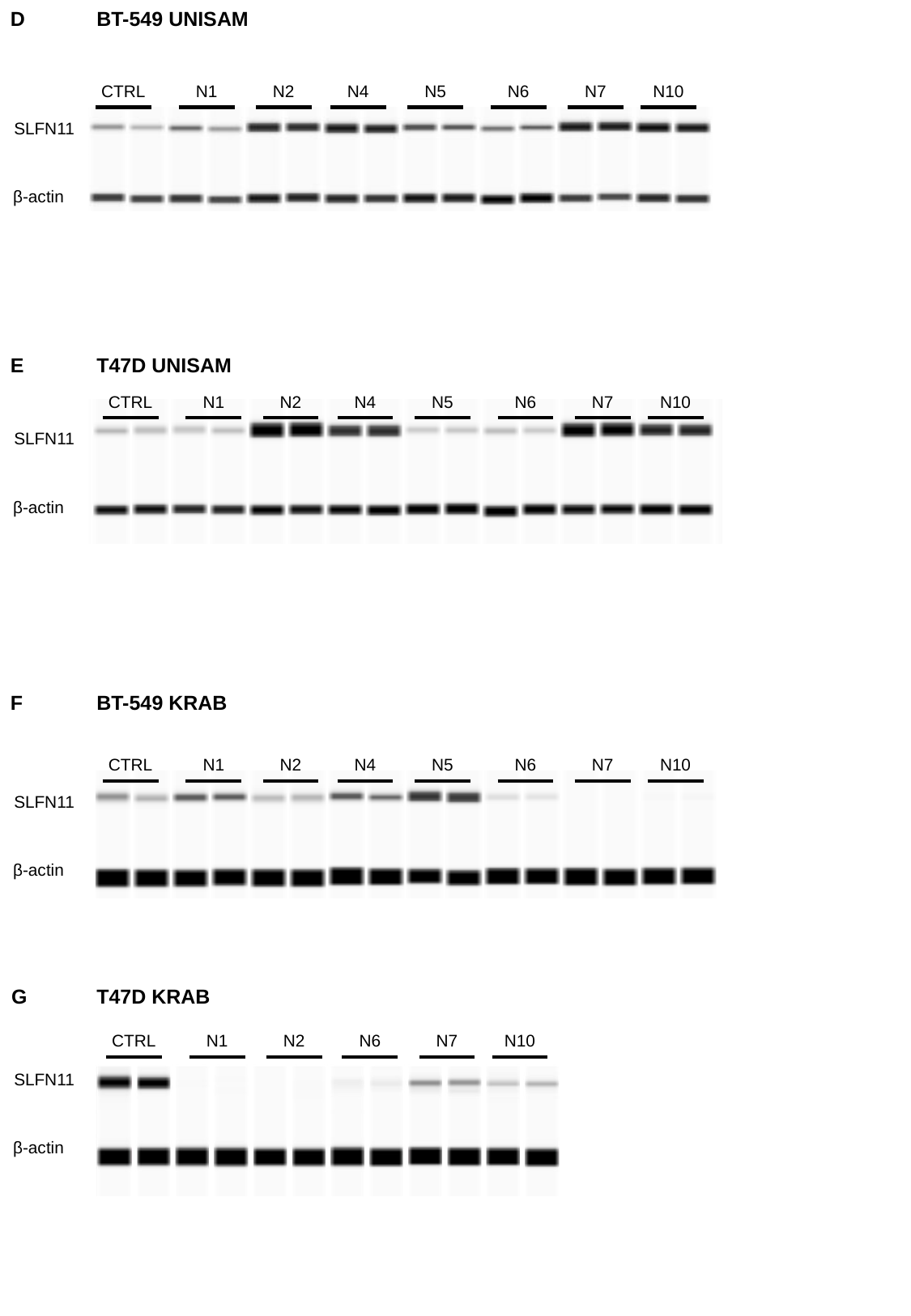

D
BT-549 UNISAM
CTRL
N1
N2
N4
N5
N6
N7
N10
SLFN11
β-actin
E
T47D UNISAM
CTRL
N1
N2
N4
N5
N6
N7
N10
SLFN11
β-actin
F
BT-549 KRAB
CTRL
N1
N2
N4
N5
N6
N7
N10
SLFN11
β-actin
G
T47D KRAB
CTRL
N1
N2
N6
N7
N10
SLFN11
β-actin

## Slide 5
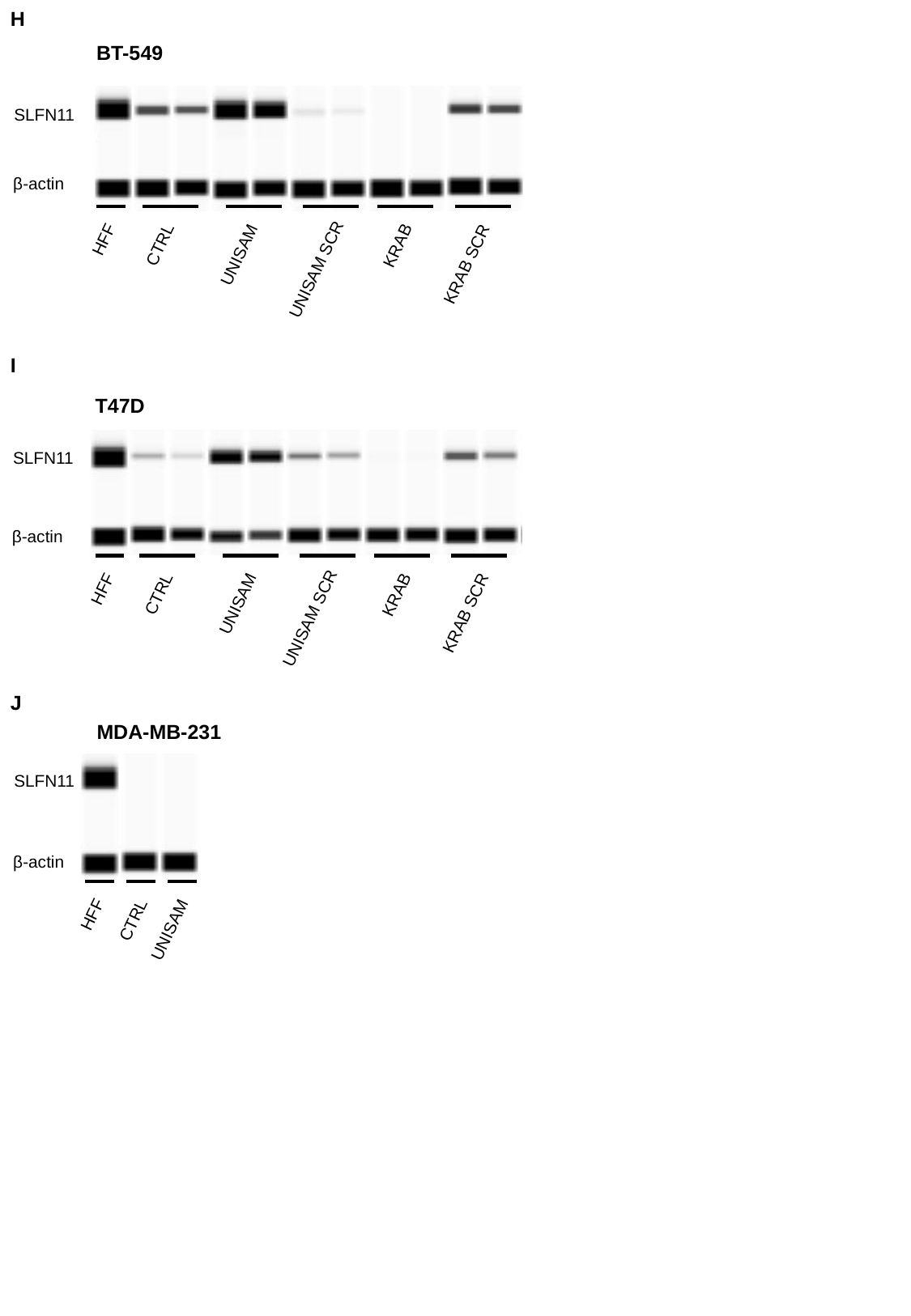

H
BT-549
SLFN11
β-actin
HFF
CTRL
KRAB
UNISAM
KRAB SCR
UNISAM SCR
I
T47D
SLFN11
β-actin
HFF
CTRL
KRAB
UNISAM
KRAB SCR
UNISAM SCR
J
MDA-MB-231
SLFN11
β-actin
HFF
CTRL
UNISAM

## Slide 6
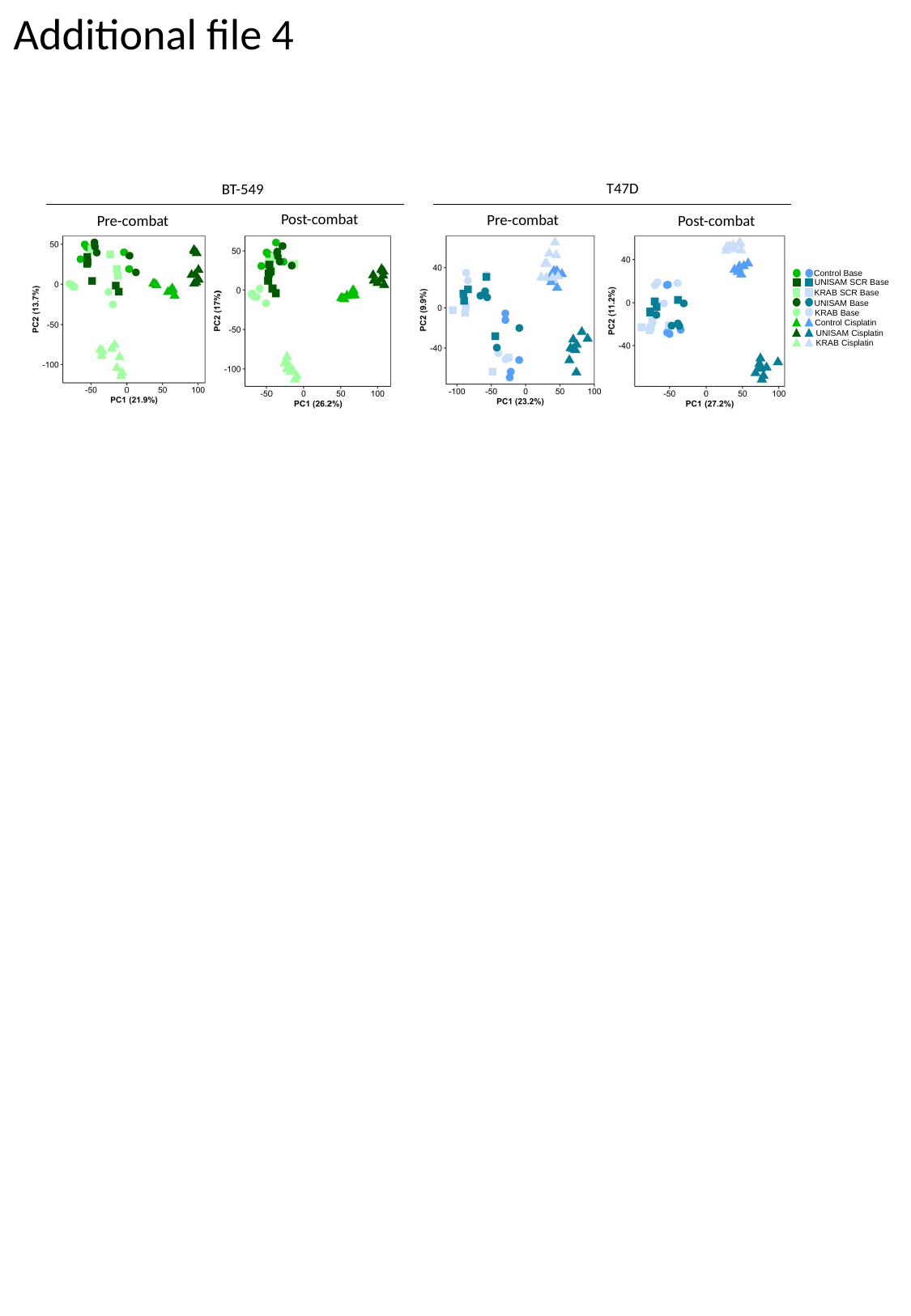

Additional file 4
T47D
BT-549
Post-combat
Pre-combat
Pre-combat
Post-combat
Control Base
UNISAM SCR Base
KRAB SCR Base
UNISAM Base
KRAB Base
UNISAM Cisplatin
KRAB Cisplatin
Control Cisplatin

## Slide 7
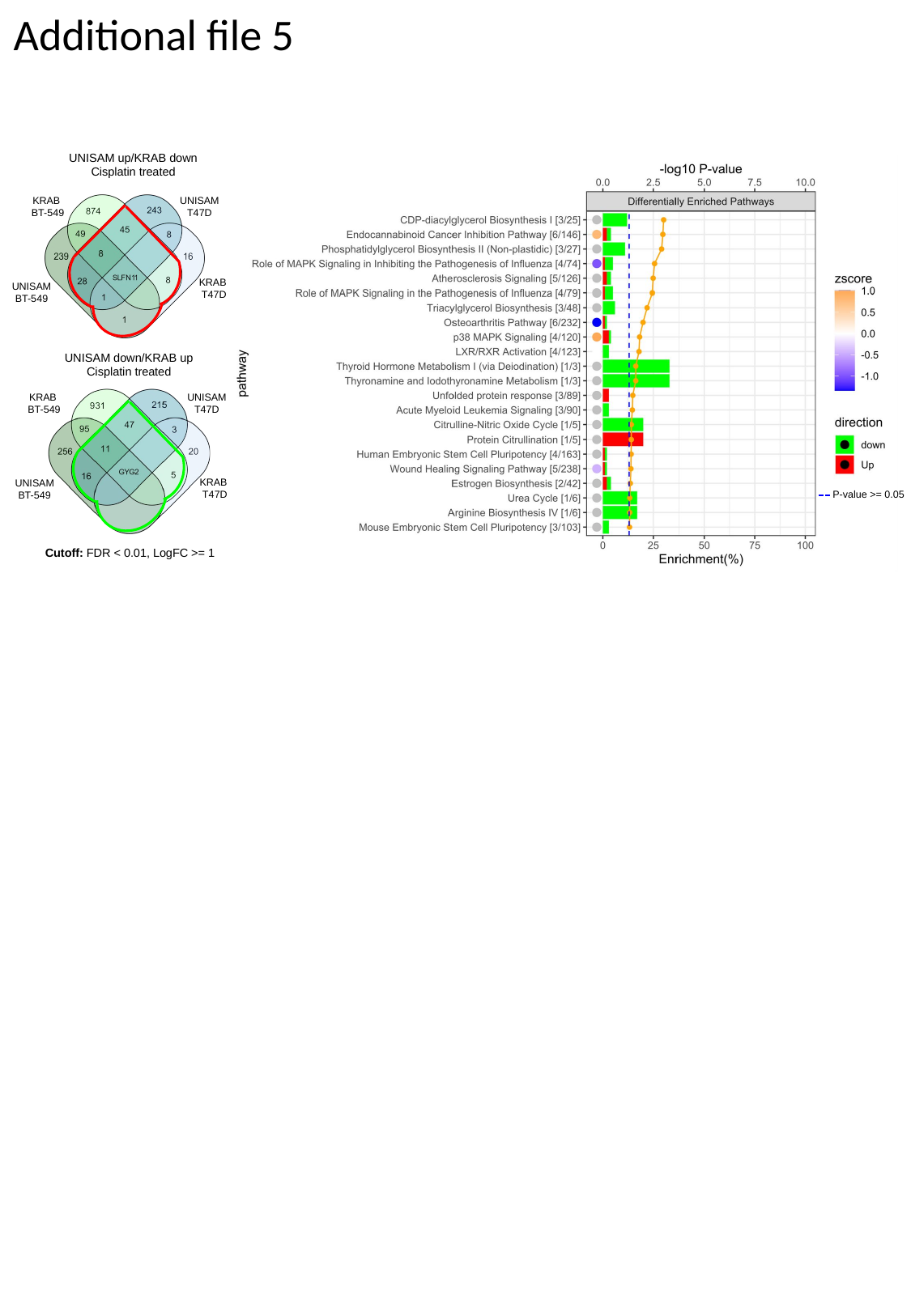

Additional file 5
UNISAM up/KRAB down
Cisplatin treated
UNISAM T47D
KRAB
 BT-549
KRAB
T47D
UNISAM BT-549
UNISAM down/KRAB up
Cisplatin treated
UNISAM T47D
KRAB
 BT-549
KRAB
T47D
UNISAM BT-549
P-value >= 0.05
Cutoff: FDR < 0.01, LogFC >= 1
